# Supplementary material for: Epidemiology and excess mortality of antimicrobial resistance in bacteraemias among cancer patients: a cohort study using routinely collected health data from regional hospital trusts in Oxford and Oslo, 2008–2018
Source: BMJ Open. 2025 Jun 13;15(6):e092740. doi: 10.1136/bmjopen-2024-092740 (PMC12164652; doi:10.1136/bmjopen-2024-092740)
Supplement: Supplementary Table 1 [file bmjopen-15-6-s001.docx]

## Supplementary material

These are the supplementary material to the article “Epidemiology and excess mortality of antimicrobial resistance in bacteraemias among cancer patients: a cohort study using routinely collected health data from regional hospital trusts in Oxford and Oslo, 2008-2018” by authors Anders Skyrud Danielsen, Cherry Lim, Chang Ho Yoon, Jon Michael Gran, Oliver Kacelnik, David W. Eyre, and Jørgen Vildershøj Bjørnholt.

The corresponding author is Anders Skyrud Danielsen, [andersskyrud.danielsen@fhi.no](mailto:andersskyrud.danielsen@fhi.no).

Table S1. Characteristics of cancer patients diagnosed in 2008-2018 and followed three years after their first cancer diagnosis at Oxford University Hospitals and Oslo University Hospital, categorised by the antimicrobial resistance phenotype of their first bacteraemia episode in the 30-day follow-up period. Continuous covariates are presented with median and interquartile range, while categorical covariates are presented with frequencies and percentages.

|  | **Oxford** | | | | | **Oslo** | | | | |
| --- | --- | --- | --- | --- | --- | --- | --- | --- | --- | --- |
|  | **Overall** | **Susceptible** | | **Resistant** | | **Overall** | **Susceptible** | | **Resistant** | |
| **Characteristics** | **1929** | n = | 1620 | n = | 309 | **1640** | n = | 1541 | n = | 99 |
| **All-cause 30-day mortality** | **509** | 404 | (25%) | 105 | (34%) | **346** | 334 | (22%) | 12* | (12%) |
| **Age (continuous)** | **69 (57, 79)** | 70 | (58, 79) | 66 | (55, 76) | **66 (54, 75)** | 66 | (55, 75) | 59 | (44, 68) |
| **Age (categorical)** |  |  |  |  |  |  |  |  |  |  |
| ≤39 years | **175** | 139 | (9%) | 36 | (12%) | **171** | 151 | (10%) | 20 | (20%) |
| 40-49 years | **125** | 103 | (6%) | 22 | (7%) | **140** | 126 | (8%) | 14 | (14%) |
| 50-59 years | **248** | 199 | (12%) | 49 | (16%) | **256** | 239 | (16%) | 17 | (17%) |
| 60-69 years | **432** | 355 | (22%) | 77 | (25%) | **441** | 416 | (27%) | 25 | (25%) |
| 70-79 years | **497** | 428 | (26%) | 69 | (22%) | **372** | 356 | (23%) | 16 | (16%) |
| ≥80 years | **452** | 396 | (24%) | 56 | (18%) | **260** | 253 | (16%) | 7 | (7%) |
| **Sex** |  |  |  |  |  |  |  |  |  |  |
| F | **719** | 624 | (39%) | 95 | (31%) | **657** | 621 | (40%) | 36 | (36%) |
| M | **1210** | 996 | (61%) | 214 | (69%) | **983** | 920 | (60%) | 63 | (64%) |
| **Cancer type and treatment received** |  |  |  |  |  |  |  |  |  |  |
| Lymphoid or haematopoietic, no transplant | **442** | 358 | (22%) | 84 | (27%) | **271** | 259 | (17%) | 12 | (12%) |
| Lymphoid or haematopoietic, transplant | **244** | 190 | (12%) | 54 | (17%) | **204** | 186 | (12%) | 18 | (18%) |
| Solid, other or unknown, no surgery | **173** | 153 | (9%) | 20 | (6%) | **256** | 240 | (16%) | 16 | (16%) |
| Solid, other or unknown, surgery | **1070** | 919 | (57%) | 151 | (49%) | **909** | 856 | (56%) | 53 | (54%) |
| **Year of cancer diagnosis** |  |  |  |  |  |  |  |  |  |  |
| 2008 | **227** | 186 | (11%) | 41 | (13%) | **73** | 66 | (4%) | 7 | (7%) |
| 2009 | **163** | 144 | (9%) | 19 | (6%) | **109** | 102 | (7%) | 7 | (7%) |
| 2010 | **169** | 143 | (9%) | 26 | (8%) | **156** | 144 | (9%) | 12 | (12%) |
| 2011 | **148** | 111 | (7%) | 37 | (12%) | **154** | 147 | (10%) | 7 | (7%) |
| 2012 | **152** | 116 | (7%) | 36 | (12%) | **144** | 139 | (9%) | 5 | (5%) |
| 2013 | **174** | 154 | (10%) | 20 | (6%) | **180** | 164 | (11%) | 16 | (16%) |
| 2014 | **165** | 136 | (8%) | 29 | (9%) | **163** | 157 | (10%) | 6 | (6%) |
| 2015 | **169** | 142 | (9%) | 27 | (9%) | **146** | 135 | (9%) | 11 | (11%) |
| 2016 | **191** | 169 | (10%) | 22 | (7%) | **192** | 181 | (12%) | 11 | (11%) |
| 2017 | **176** | 150 | (9%) | 26 | (8%) | **157** | 150 | (10%) | 7 | (7%) |
| 2018 | **195** | 169 | (10%) | 26 | (8%) | **166** | 156 | (10%) | 10 | (10%) |
| **Other primary infection** | **941** | 810 | (50%) | 131 | (42%) | **690** | 649 | (42%) | 41 | (41%) |
| **Polymicrobial bacteraemia** | **76** | 63 | (4%) | 13 | (4%) | **248** | 226 | (15%) | 22 | (22%) |
| **Charlson Comorbidity Index** |  |  |  |  |  |  |  |  |  |  |
| ≤2 | **807** | 683 | (42%) | 124 | (40%) | **586** | 549 | (36%) | 37 | (37%) |
| 3-5 | **591** | 490 | (30%) | 101 | (33%) | **409** | 383 | (25%) | 26 | (26%) |
| ≥6 | **531** | 447 | (28%) | 84 | (27%) | **645** | 609 | (40%) | 36 | (36%) |
| **Past hospitalisation** | **19 (9, 39)** | 18 | (8, 35) | 29 | (15, 53) | **40 (21, 70)** | 40 | (21, 68) | 57 | (30, 90) |

*The number of outcome events in the Oslo data is small, threatening model stability and convergence. Hence, further analyses to estimate the excess mortality and PAF were not performed on the Oslo data set.


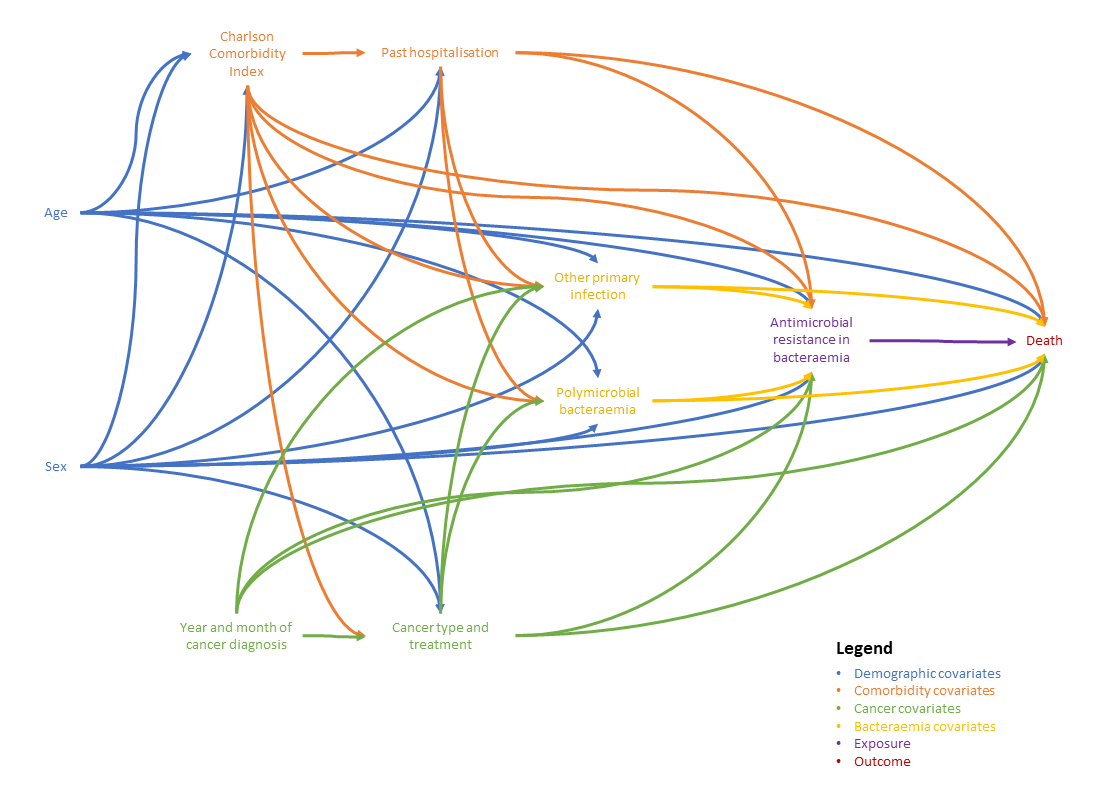


**Figure S2.** Directed acyclic graph.

**Supplementary material S3 – Detailed covariate description**

- Sex: Recorded as either female or male.
- Age: Treated as a continuous variable, derived from the date of birth.
- Charlson Comorbidity Index (CCI): Calculated from ICD-10 diagnosis codes within one year prior to the bacteraemia episode.
- Past hospitalisation: The cumulative sum of all hospital stay days within one year prior to the bacteraemia episode, reflecting previous exposure to secondary healthcare, but not to long-term care facilities or primary healthcare. “Patient hotels” are counted as inpatient stays in Norway, and the logistical challenges posed by Norway's geography combined with OsUH’s national functions might contribute to differences in hospitalisation days.
- Other infectious focus: Identified by the presence of infectious syndromes in other sites than blood (thus excluding septicaemia). As the specimen was not always known at OsUH if not blood, we relied only on ICD-10 codes at both hospitals, basing our classifications of the focus on the Clinical Classifications Software Refined [1].
- Year and month of cancer diagnosis: Used to adjust for time-related changes in cancer treatment and supportive care.
- Cancer type and treatment: Categorised based on malignancy and treatment within a year before follow-up, including solid cancers with or without surgery and haematological cancers with or without stem cell transplantation. To classify surgeries, a concept-based approach was adopted in which a list of common terms for surgeries in both English and Norwegian were used to search in the procedure code descriptions. To capture exposures related to transplantation, a 90-day lookahead was also adopted, as transplantations were sometimes planned with (myeloablative and/or immunosuppressive) conditioning started, without the procedure having been completed. This coding was not perfect, however, due to immortal time bias.
- Polymicrobial bacteraemia: A binary variable defined by the identification of different causative agents in the same or adjacent days' blood cultures, with coagulase-negative staphylococci generally considered contamination. Please note that differences in case mix, including OsUH's role as Norway's specialist cancer centre, may account for variations in patient age and polymicrobial bacteraemias due to diverse underlying immunosuppression, among other undetermined factors.

All information from OxUH comes from the hospital's electronic health record system, coded and stored in the Infections in Oxfordshire Research Database. Sex, age, date of cancer diagnosis and cancer type at OsUH was collected from the Cancer Registry of Norway, past hospitalisation, ICD-10 codes, and procedure codes were collected from the Norwegian Patient Registry, and all microbiological data were collected from the raw data of the microbiological laboratory information systems.

**Supplementary material S4 – Detailed statistical methods**

We consider the excess mortality due to AMR to be the difference between the actual observed mortality and the expected mortality at the end of follow-up had all bacteraemia been caused by susceptible bacteria. As the latter is a counterfactual number that is not observed directly and needs to be estimated, excess mortality is most precisely defined using counterfactual notation. The resulting estimand may be described as

$$Excess mortality due to AMR= Pr\left( Y=1 \right)-Pr(Y^{A=0}=1),$$

where $Y$ is the outcome variable indicating mortality, with $Y=1$ representing death and $Y=0$ indicating survival at the end of follow-up. $A$ represents the exposure, with $A=1$ indicating a bacteraemia with a resistant phenotype and $A=0$ indicating a susceptible phenotype. Thus, $Pr\left( Y=1 \right)$represents the observed mortality in the population, and $Pr(Y^{A=0}=1)$ represents the expected mortality if all participants had a bacteraemia caused by a susceptible phenotype. We will also estimate the population-attributable fraction (PAF), which we define as

$$PAF due to AMR=\frac{Pr\left( Y=1 \right)-Pr(Y^{A=0}=1),}{Pr\left( Y=1 \right)}.$$

PAF is inconsistently defined in epidemiological literature [2], our definition was based on our definition of excess mortality and consisted of contrasting the observed and the expected mortalities at the end of follow-up.

To estimate the expected mortality in the absence of AMR, i.e. $Pr\left( Y^{A=0}=1 \right)$, we weighted the bacteraemias without AMR by the inverse probability of not having AMR, conditioning on the pre-identified confounders [3,4]. Under the mentioned identification assumptions, this weighted subpopulation is representative of a full population without AMR. We calculated stabilised, untruncated inverse probability weights using logistic regression, with the confounders as independent variables and the exposure group as the dependent variable. Covariates were included as outlined in the methods chapter. Continuous variables were modelled linearly, as more complex functional forms offered only marginal improvements in model fit based on Akaike’s information criterion. The expected cumulative incidence function of mortality was then subtracted from the observed cumulative incidence function to obtain the excess mortality estimate. We then used nonparametric bootstrapping with 1000 bootstrap samples to calculate percentile-based 95% confidence intervals for both excess mortality and PAF [3]. This approach required a certain sample size as insufficient events in both groups to ensure model stability and convergence may render bootstrapping and estimation procedures unfeasible.

We then estimated the unweighted Kaplan-Meier survival curves by exposure groups, and the unweighted survival curve for the overall observed mortality with the weighted survival curve for the counterfactual scenario with confidence intervals bootstrapped at each time point. This would correspond to defining excess mortality as the difference between survival functions $S\left( t \right)-S^{A=0}(t)$ effectively quantifying it day-by-day in our weighted analysis. Density plots then illustrated the excess mortality and PAF at the end of follow-up with the bootstrapped confidence intervals. Excess mortality was also estimated for each drug-pathogen combination separately and a sensitivity analysis was performed for the OxUH data adding a linear term for cumulative days on antibiotics in the previous 365 days. Lastly, the E-value was calculated to evaluate the minimum strength of association required for an unmeasured confounder to fully explain away the estimated excess mortality due to AMR, under the assumption that measured covariates were correctly controlled for; this was done by first converting the risk difference to a risk ratio by dividing the counterfactual and excess mortality by the counterfactual mortality [5].

A causal interpretation of the estimates in our study relies on three central identifiability conditions being met: consistency, exchangeability, and positivity [3].

1. **Consistency:** This condition asserts that the observed outcome for an individual under their actual exposure is the same as the estimated outcome if that patient happened to be ascribed the same counterfactual exposure. For example, if a patient had a susceptible phenotype, their estimated outcome in the counterfactual scenario where all bacteraemias were caused by susceptible phenotypes should be identical to their observed outcome. This means the treatment and outcome are well-defined and consistently applied.
2. **Exchangeability:** Also known as no unmeasured confounding, this condition posits that the groups being compared are similar in all relevant respects except for the exposure of interest. It necessitates a correct adjustment set where all known confounding variables are accounted for without introducing bias by conditioning on certain covariates known as colliders. This mimics the randomisation in controlled trials. In our context, we assume that by controlling for all identified confounders (as informed by our DAG), the exposure groups (resistant vs. susceptible phenotypes) are exchangeable. However, unmeasured confounding remains a concern, as it is challenging to be certain that our DAG is completely accurate. Sensitivity analyses and the calculation of the E-value, which assesses the robustness of our findings to potential unmeasured confounders, address this limitation such that it can be assessed whether the assumption of exchangeability is reasonable.
3. **Positivity:** This condition assumes that every individual has a non-zero probability of receiving the exposure within each level of every included covariate. This is crucial to avoid biases in effect estimation due to a limited data range. In our study, positivity was ensured by the selection of the patient population and the coding of covariates, such as combining cancer types and treatments. We ensured there were both resistant and susceptible phenotypes across all levels of our covariates, reducing the risk of violating this assumption.

**Table S5.** The unweighted and weighted mean values of the continuous and dichotomous covariates included in the propensity score model used to weight the population in the counterfactual scenario of absence of antimicrobial resistance in all bacteraemias among cancer patients at Oxford University Hospitals, 2008-2018.

|  | **Mean values** | |
| --- | --- | --- |
|  | **AMR absent** | **AMR present** |
| **Unweighted** |  |  |
| Age | 66.23 | 62.95 |
| Sex | 0.61 | 0.69 |
| Other primary | 0.50 | 0.42 |
| Polymicrobial | 0.04 | 0.04 |
| Previous hospitalisation | 27.04 | 41.47 |
| **Weighted** |  |  |
| Age | 65.74 | 65.26 |
| Sex | 0.63 | 0.65 |
| Other primary | 0.49 | 0.50 |
| Polymicrobial | 0.04 | 0.04 |
| Previous hospitalisation | 29.60 | 30.58 |

**Table S6.** Excess mortality estimates and bootstrapped 95% confidence intervals of head-to-head comparisons of the key drug-pathogen combinations isolated from the blood of cancer patients diagnosed in 2008-2018 at Oxford University Hospitals.

|  | **Excess mortality** | | |
| --- | --- | --- | --- |
|  | **Point estimate** | **Lower 95% confidence limit** | **Upper 95% confidence limit** |
| *Escherichia coli*, third-generation cephalosporin-resistant | 0.6 % | -0.4 % | 1.6 % |
| *Staphylococcus aureus*, methicillin-resistant | 2.6 % | -0.9 % | 5.9 % |
| *Pseudomonas aeruginosa*, carbapenem-resistant | 1.9 % | -5.7 % | 10.5 % |
| *Klebsiella pneumoniae*, third-generation cephalosporin-resistant | 1.8 % | -2.3 % | 5.8 % |
| *Acinetobacter* spp., carbapenem-resistant | -1.7 % | -4.3 % | 0.0 % |
| *Klebsiella pneumoniae*, carbapenem-resistant | 1.9 % | -1.9 % | 5.9 % |
| Enterococci, vancomycin-resistant | 3.1 % | -2.2 % | 8.5 % |
| *Escherichia coli*, carbapenem-resistant | 0.6 % | -0.6 % | 1.7 % |

**Table S7.** Cumulative antibiogram of the included microbes isolated from the blood of cancer patients diagnosed in 2008-2018 at Oxford University Hospitals and Oslo University Hospital. Both laboratories used cefoxitin to screen for methicillin resistance in S. aureus. However, due to differences in local laboratory workflows and reporting practices, cefoxitin results are shown for Oslo, whereas flucloxacillin is presented for Oxford.

|  | **Oxford** | | **Oslo** | |
| --- | --- | --- | --- | --- |
|  | **Tested (n)** | **Resistant (%)** | **Tested (n)** | **Resistant (%)** |
| **Escherichia coli** |  |  |  |  |
| Amoxicillin | 925 | 61.4 | - | - |
| Amoxicillin/clavulanate | 934 | 38.3 | 222 | 27.9 |
| Ampicillin | - | - | 784 | 49.1 |
| Piperacillin/tazobactam | 933 | 8.5 | 785 | 5.5 |
| Ceftriaxone | 934 | 8.8 | - | - |
| Cefotaxime | - | - | 776 | 7.9 |
| Ceftazidime | 925 | 7.8 | 770 | 4.8 |
| Meropenem | 934 | 0.3 | 329 | 0.0 |
| Ciprofloxacin | 934 | 13.9 | 774 | 15.0 |
| Gentamicin | 934 | 6.6 | 772 | 8.5 |
| Trimethoprim-sulfamethoxazole | 570 | 29.8 | 341 | 42.2 |
| **Klebsiella pneumoniae** |  |  |  |  |
| Amoxicillin/clavulanate | 223 | 26.9 | 78 | 11.5 |
| Piperacillin/tazobactam | 224 | 12.5 | 240 | 5.0 |
| Ceftriaxone | 223 | 14.3 | - | - |
| Cefotaxime | - | - | 239 | 5.4 |
| Ceftazidime | 221 | 14.0 | 236 | 5.1 |
| Meropenem | 225 | 0.0 | 111 | 0.0 |
| Ciprofloxacin | 225 | 12.4 | 240 | 7.1 |
| Gentamicin | 225 | 10.7 | 240 | 4.2 |
| Trimethoprim-sulfamethoxazole | 138 | 19.6 | 112 | 19.6 |
| **Pseudomonas aeruginosa** |  |  |  |  |
| Piperacillin/tazobactam | 239 | 4.2 | 64 | 14.1 |
| Ceftazidime | 235 | 2.6 | 62 | 9.7 |
| Meropenem | 238 | 4.2 | 53 | 5.7 |
| Amikacin | 124 | 0.0 | - | - |
| Gentamicin | 239 | 0.4 | 61 | 6.6 |
| Ciprofloxacin | 239 | 3.3 | 62 | 4.8 |
| Colistin | 96 | 3.1 | - | - |
| **Acinetobacter spp.** |  |  |  |  |
| Meropenem | 34 | 2.9 | 5 | 0.0 |
| Colistin | 9 | 0.0 | - | - |
| Ciprofloxacin | 34 | 2.9 | 6 | 0.0 |
| **Staphylococcus aureus** |  |  |  |  |
| Flucloxacillin | 300 | 13.7 | - | - |
| Cefoxitin | - | - | 271 | 3.7 |
| Vancomycin | 299 | 0.0 | 102 | 0.0 |
| Clindamycin | 144 | 11.1 | 287 | 5.6 |
| Erythromycin | 299 | 15.1 | 286 | 6.6 |
| Linezolid | 147 | 1.4 | 97 | 4.1 |
| Trimetoprim-sulfamethoxazole | 141 | 3.5 | 98 | 5.1 |
| **Enterococci** |  |  |  |  |
| Ampicillin | 48 | 68.8 | 342 | 46.2 |
| Amoxicillin | 231 | 55.4 | - | - |
| Vancomycin | 246 | 24.4 | 338 | 1.5 |
| Linezolid | 177 | 1.7 | 328 | 0.6 |
| Daptomycin | 70 | 2.9 | - | - |

**References**

[1] Agency for Healthcare Research and Quality. Clinical Classifications Software Refined (CCSR) for ICD-10-CM Diagnoses n.d. https://hcup-us.ahrq.gov/toolssoftware/ccsr/dxccsr.jsp (accessed December 11, 2023).

[2] Cube M von, Timsit J-F, Schumacher M, Motschall E, Schumacher M. Quantification and interpretation of attributable mortality in core clinical infectious disease journals. Lancet Infect Dis 2020;20:e299–306. https://doi.org/10.1016/S1473-3099(20)30485-0.

[3] Hernán MA, Robins JM. Causal Inference: What If. Boca Raton: Chapman & Hall/CRC; 2020.

[4] HUBBARD AE, LAAN MJVD. Population intervention models in causal inference. Biometrika 2008;95:35–47.

[5] VanderWeele TJ, Ding P. Sensitivity Analysis in Observational Research: Introducing the E-Value. Ann Intern Med 2017;167:268–74. https://doi.org/10.7326/M16-2607.
