# Supplementary material for: Epidemiology and excess mortality of antimicrobial resistance in bacteraemias among cancer patients: a cohort study using routinely collected health data from regional hospital trusts in Oxford and Oslo, 2008–2018
Source: BMJ Open. 2025 Jun 13;15(6):e092740. doi: 10.1136/bmjopen-2024-092740 (PMC12164652; doi:10.1136/bmjopen-2024-092740)
Supplement: Supplementary file 2 [file bmjopen-2024-092740.reviewer_comments.pdf]

PEER REVIEW HISTORY

BMJ Open publishes all reviews undertaken for accepted manuscripts. Reviewers are asked to complete a checklist review form and are provided with free text boxes to elaborate on their assessment. These free text comments are reproduced below.

ARTICLE DETAILS

Title (Provisional)

Epidemiology and excess mortality of antimicrobial resistance in bacteraemias among cancer patients: a cohort study using routinely collected health data from regional hospital trusts in Oxford and Oslo, 2008-2018

Authors

Danielsen, Anders Skyrud; Lim, Cherry; Yoon, Chang Ho; Gran, Jon Michael; Kacelnik, Oliver; Eyre, David; Bjornholt, Jorgen Vildershoj

---

VERSION 1 - REVIEW

**Reviewer**	**1**
**Name**	**Amanati, Ali**
**Affiliation**	**Shiraz University of Medical Sciences**
**Date**	**29-Nov-2024**
**COI**	**None**

While the time-fixed counterfactual framework is a powerful methodological approach used in this study some significant objections need your consideration:

--It assumes that past outcomes do not influence current treatment assignments. If this assumption does not hold—such as when prior health status affects the likelihood of receiving certain treatments—the estimates may be biased. For example, more critically ill patients are more likely to receive MDRO treatment due to their severe condition, so simply comparing outcomes without accounting for this could lead to an overestimation of the effect of AMR on mortality.

--The framework is sensitive to measurement errors in both treatment and outcome variables. If there are inaccuracies in recording whether an infection is resistant or if mortality is correctly attributed to AMR, it can skew results. For example, if a patient dies from complications unrelated to the infection but is recorded as having died from an MDRO infection, it could inflate the estimated excess mortality due to AMR.

--The framework may struggle to account for dynamic treatment effects where the impact of treatment varies over time. For instance, if the effectiveness of treatments changes as new

therapies are introduced or as resistance patterns evolve, this variability can complicate causal inference.

--Unobserved time-varying confounders can complicate causal inference. If there are factors that change over time and affect both treatment and outcome but are not measured, they can introduce bias into the estimated treatment effects. For example, changes in hospital protocols for treating bacteremia over time could affect outcomes but may not be captured in the analysis.

Other minor concerns:

--The study highlights that only about half of the studies assessing AMR's impact on mortality report increased mortality, indicating that results may vary significantly based on the prevalence of AMR in different settings. This variability suggests that direct comparisons with other studies may be challenging, which could lead to potential misinterpretations of the results.

**Reviewer**	**2**
**Name**	**Baltas , Ioannis**
**Affiliation**	**University College London Hospitals NHS Foundation Trust**
**Date**	**25-Jan-2025**
**COI**	**None**

This retrospective cohort study in two large hospitals in the UK and Norway investigated the excess mortality from AMR among 8 important pathogen-drug combinations in patients with cancer and bacteraemia. This topic should of interest to the readers of BMJ Open and aids ongoing global efforts to quantify the impact of resistant infections on mortality. The authors have presented their results clearly and the article is very well written. This study should eventually be published but I suggest the following changes that would improve its overall quality:

Lack of adjustment for in vitro effective therapy: The authors correctly identify in the discussion section that “the impact of AMR on mortality is thus almost exclusively indirect and mediated through the administration of active or inactive antibiotic treatment”. Yet, they do not include in vitro effective therapy within 24h for example as a key variable in their DAG. Given this is a registry study, I appreciate that they will likely not have access to this data. In my experience, in the UK setting most cancer patients will be treated empirically with an anti-pseudomonal penicillin, anti-pseudomonal cephalosporin or carbapenem with or without an aminoglycoside. This will likely be effective for many of the infections selected by the authors (at least the Gram-negatives ones). Local guidelines/screen might also suggest empirical MRSA coverage. The AMR attributable mortality of a cohort with a high

probability of effective empirical treatment will be a lot lower than of the same cohort with lower probability (due to lack of access to effective drugs as in LMIC, higher AMR rates like in Southern Europe, or inappropriate empirical guidelines in less resources settings). I suggest the authors provide a description of the empirical treatment regimens in both centers over the years to add context, reflect further in their discussion section on this limitation and clearly state in their abstract and discussion that these results likely apply only to high income settings with low levels of AMR and high likelihood of effective empirical treatment. Despite all these factors, that would reduce the effect size of AMR on mortality, it is still very interesting that the authors find an effect on mortality.

Polymicrobial bacteraemias: These can represent a very diverse group of conditions, increasing the chance of unmeasured confounding in an observational study. Given the lack of detailed patient-level data, I would suggest excluding those from the analysis completely or as a minimum perform a sensitivity analysis.

Length of stay: The authors noted a significant increase in length of stay in hospital in patients with resistant infections in univariable analysis. It would be interesting to see whether there was an association as well in multivariable analysis. Frailty would be a key confounder to adjust in the analysis if available, but currently presented variables might suffice.

Antibiograms: The authors should provide detailed antibiograms for all the bacteraemias included in the study (per species) in order to help add context to the local epidemiology of this study.

Type of malignancy: The authors have analysed solid and haematological malignancies together. These patient groups are significantly different and a subgroup analysis should be performed.

VERSION 1 - AUTHOR RESPONSE

Reviewer: 1
Prof. Ali Amanati, Shiraz University of Medical Sciences
Comments to the Author:
While the time-fixed counterfactual framework is a powerful methodological approach used in this study some significant objections need your consideration:

We thank the reviewer for their assessment.

--It assumes that past outcomes do not influence current treatment assignments. If this assumption does not hold—such as when prior health status affects the likelihood of receiving certain treatments—the estimates may be biased. For example, more critically ill patients are more likely to receive MDRO treatment due to their severe condition, so simply comparing outcomes without accounting for this could lead to an overestimation of the effect of AMR on mortality.

This is a fair concern, and although we do not include other covariates than whether the bacteraemia is polymicrobial as an indicator of severity of the bloodstream infection, the prior health status is captured in several of the included covariates – such as the Charlson Comorbidity Index, past hospitalisations, and cancer type/treatment. These covariates are thought to capture any possible confounding by indication introduced by the treatment given against the bacteraemia. We have clarified that in the covariate section of the Methods chapter:

"[Charlson Comorbidity Index (CCI): Calculated from ICD-10 diagnosis codes within one year prior to the bacteraemia episode, reflecting the burden of comorbid conditions] **and severity of illness, which may influence the likelihood of receiving broad-spectrum antibiotics.**"

--The framework is sensitive to measurement errors in both treatment and outcome variables. If there are inaccuracies in recording whether an infection is resistant or if mortality is correctly attributed to AMR, it can skew results. For example, if a patient dies from complications unrelated to the infection but is recorded as having died from an MDRO infection, it could inflate the estimated excess mortality due to AMR.

While misclassification is a general concern in all studies using routinely collected health data, our study does not rely on cause-specific mortality attribution, but rather on all-cause mortality across exposure groups. Therefore, misclassification of the cause of death is not a source of bias in our analysis. With regard to exposure misclassification, the AMR phenotype was defined based on antimicrobial susceptibility testing directly extracted from hospital laboratory information systems. While errors cannot be ruled out, we believe the risk of substantial misclassification is low given the automated nature of data capture. Nonetheless, we now acknowledge this as a limitation in the revised manuscript directly before the conclusion:

"While we cannot rule out some misclassification of resistance status or mortality, the use of structured EHR and laboratory data minimises this risk. Any remaining misclassification is likely non-differential and would bias estimates towards the null."

--The framework may struggle to account for dynamic treatment effects where the impact of treatment varies over time. For instance, if the effectiveness of treatments changes as new therapies are introduced or as resistance patterns evolve, this variability can complicate causal inference.

Our time-fixed modelling approach was selected for its transparency and interpretability, acknowledging that it simplifies the complexity of dynamic clinical care. However, secular trends in cancer treatment and resistance epidemiology are accounted for in our adjustment set via the inclusion of year and month of cancer diagnosis. This variable helps control for changes in treatment protocols and broader shifts in the healthcare context over the study period. We have expand slightly on its description now:

"Year and month of cancer diagnosis: Used to adjust for secular trends, including changes in cancer treatment, resistance patterns, and supportive (sepsis) care over time."

--Unobserved time-varying confounders can complicate causal inference. If there are factors that change over time and affect both treatment and outcome but are not measured, they can introduce bias into the estimated treatment effects. For example, changes in hospital protocols for treating bacteremia over time could affect outcomes but may not be captured in the analysis.

"Our study was designed using a time-fixed framework, which assumes fixed values for exposure and covariates at baseline. We adopted this approach deliberately, prioritising interpretability and robustness over modelling complexity. Patients were followed for a short time period (30 days), during which substantial changes to treatment protocols or resistance profiles are unlikely. To account for broader temporal trends in treatment practices and resistance patterns, we included calendar year and month of cancer diagnosis as covariates. As noted in the manuscript, our "time-fixed counterfactual framework represents a simplification of complex clinical realities" (Discussion), and potential unmeasured confounding remains a limitation, for which we have assessed robustness using the E-value."

Other minor concerns:
--The study highlights that only about half of the studies assessing AMR's impact on mortality report increased mortality, indicating that results may vary significantly based on the prevalence of AMR in different settings. This variability suggests that direct comparisons with other studies may be challenging, which could lead to potential misinterpretations of the results.

We agree that direct comparisons across studies can be challenging due to substantial variability in AMR prevalence, patient populations, and settings. This point is already discussed in the manuscript, but we have now added a brief clarification to emphasise the limitation in comparing our results directly with those of other studies.

"[Only about half of studies assessing the impact of AMR on mortality report increased mortality, and mainly from settings with a high prevalence of AMR, yet many fail to find significant impacts.] **This variation highlights the limited comparability across studies conducted in different contexts.** [The difficulties may be due to residual confounding or small sample sizes], **either due to low prevalence or** [a focus on specific infectious syndromes or drug-pathogen combinations, challenges that our study also potentially faces] [5,25–27].

Reviewer: 2
Dr. Ioannis Baltas , University College London Hospitals NHS Foundation Trust
Comments to the Author:
This retrospective cohort study in two large hospitals in the UK and Norway investigated the excess mortality from AMR among 8 important pathogen-drug combinations in patients with cancer and bacteraemia. This topic should of interest to the readers of BMJ Open and aids ongoing global efforts to quantify the impact of resistant infections on mortality. The authors have presented their results clearly and the article is very well written. This study should eventually be published but I suggest the following changes that would improve its overall quality:

Thank you to the reviewer for their positive and constructive feedback, and for recognising the clarity and relevance of our study.

Lack of adjustment for in vitro effective therapy: The authors correctly identify in the discussion section that “the impact of AMR on mortality is thus almost exclusively indirect and mediated through the administration of active or inactive antibiotic treatment”. Yet, they do not include in vitro effective therapy within 24h for example as a key variable in their DAG. Given this is a registry study, I appreciate that they will likely not have access to this data. In my experience, in the UK setting most cancer patients will be treated empirically with an anti-pseudomonal penicillin, anti-pseudomonal cephalosporin or carbapenem with or without an aminoglycoside. This will likely be effective for many of the infections selected by the authors (at least the Gram-negatives ones). Local guidelines/screen might also suggest empirical MRSA coverage. The AMR attributable mortality of a cohort with a high probability of effective empirical treatment will be a lot lower than of the same cohort with lower probability (due to lack of access to effective drugs as in LMIC, higher AMR rates like in Southern Europe, or inappropriate empirical guidelines in less resources settings). I suggest the authors provide a description of the empirical treatment regimens in both centers over the years to add context, reflect further in their discussion section on this limitation and clearly state in their abstract and discussion that these results likely apply only to high income settings with low levels of AMR and high likelihood of effective empirical treatment. Despite all these factors, that would reduce the effect size of AMR on mortality, it is still very interesting that the authors find an effect on mortality.

We agree that the presence or absence of in vitro effective empirical treatment is likely a key mediator of the association between AMR and mortality, and we appreciate the opportunity to clarify this point further.

As correctly noted, our registry-based study did not include individual-level data on empirical antibiotic regimens or in vitro susceptibility profiles within the first 24 hours. However, we have now expanded the discussion to reflect more clearly how empirical regimens and context-specific factors may influence the indirect effect of AMR on mortality. In both study settings, standard empirical regimens for cancer patients with well-managed disease typically included amoxicillin/clavulanate and gentamicin at OxUH and benzylpenicillin and gentamicin at OsUH, while neutropenic patients were commonly treated with piperacillin/tazobactam in both locations. We have also noted that the magnitude of the indirect effect is likely influenced by the propensity to escalate treatment and laboratory turnaround times.

To reflect these considerations, we have revised the discussion and abstract as follows:

Revised Discussion section:

“It is important to note that the direct effect of AMR on mortality is likely low – although not zero – as there is overlap between AMR and increased virulence, e.g., due to successful clones or plasmids carrying linked resistance and virulence genes [28]. The impact of AMR on mortality is thus almost exclusively indirect and mediated through the administration of active or inactive antibiotic treatment [29–32]. **In both settings, standard empirical regimens differ slightly for patients with a well-managed**

underlying disease, with amoxicillin/clavulanate and gentamicin commonly used at OxUH and benzylpenicillin and gentamicin at OsUH, while neutropenic patients are often treated with piperacillin/tazobactam. The magnitude of the indirect effect will also depend on the propensity to escalate treatment and the laboratory turnaround time for identifying resistance. If patients expected to have infections by resistant microbes receive empirical treatment to which the microbes are susceptible, we would not expect to observe an increase in mortality. While resistant cases may be overlooked when they are not commonplace, this suggests the benefit of maintaining a low AMR prevalence, allowing healthcare providers to apply extra vigilance and tailored care in suspected AMR cases.

These findings are therefore most likely to be informative for settings with similarly low AMR prevalence and high likelihood of effective empirical treatment."

Revised Abstract conclusion:

"Conclusions: Despite estimating modest excess mortality, the mortality attributable to resistance in these two high-income, low-prevalence settings highlights the potential for escalation if global resistance trends continue to worsen."

Polymicrobial bacteraemias: These can represent a very diverse group of conditions, increasing the chance of unmeasured confounding in an observational study. Given the lack of detailed patient-level data, I would suggest excluding those from the analysis completely or as a minimum perform a sensitivity analysis.

Polymicrobial bacteraemias can indeed reflect heterogeneous clinical situations, which poses challenges for causal inference. As suggested, we conducted a sensitivity analysis using data from the OxUH cohort, in which we excluded all polymicrobial bacteraemias from the analysis rather than adjusting for them via a covariate in the weighting model. This exclusion led to a minimal change in the estimated excess mortality, from 0.01656 to 0.01599. Given the negligible impact on the results and our aim to keep the manuscript concise, we decided not to include this analysis in the manuscript. Due to the small number of events in the OsUH cohort, a similar sensitivity analysis was not feasible.

Length of stay: The authors noted a significant increase in length of stay in hospital in patients with resistant infections in univariable analysis. It would be interesting to see whether there was an association as well in multivariable analysis. Frailty would be a key confounder to adjust in the analysis if available, but currently presented variables might suffice.

We appreciate the interest in the observed difference in hospital days between patients with resistant and susceptible infections. However, the variable in question refers to the cumulative number of hospital days during the year prior to the bacteraemia episode, not the length of stay following it. As outlined in the methods section:
"Cumulative days of hospitalisation in the past year: Sum of all hospital stay days within one year prior to the bacteraemia episode, indicating prior exposure to secondary healthcare."

As this measure precedes the exposure, it cannot be influenced by the resistant bacteraemia itself. While we acknowledge the possibility of confounding, particularly by frailty, we have not constructed a directed acyclic graph (DAG) for the specific relationship between AMR status and prior healthcare exposure, and therefore have chosen not to include this analysis in the manuscript.

Nonetheless, to explore the reviewer's hypothesis, we conducted a simple multivariable OLS regression using past hospitalisation as the dependent variable and the main covariates from our weighting model as predictors. The coding of this variable ranged from 1 to 274 days, with a median of 19 and a mean of 29.4 days. This exploratory analysis suggested that AMR cases had on average 11 more hospital days in the previous year (coef = 11.4, SE = 1.89, $p < 0.001$). However, as this analysis was not pre-specified and lacks proper causal framing, we have chosen not to include it in the manuscript.

Antibiograms: The authors should provide detailed antibiograms for all the bacteraemias included in the study (per species) in order to help add context to the local epidemiology of this study.

We thank the reviewer for this helpful suggestion. A detailed cumulative antibiogram has now been added as Supplementary material S5, making it easier to appreciate the differences in local epidemiology between the two settings.

We have added the following sentence to the Study design and cohort selection section of the Methods chapter to describe this addition:

"A cumulative antibiogram was also constructed for each included microorganism by assessing resistance across a range of antimicrobials in all microbiological sources."

We have also added the following sentence to the Results chapter:

"A detailed cumulative antibiogram describing the distribution of resistance per pathogen and antimicrobial agent is provided in Supplementary material S5."

Type of malignancy: The authors have analysed solid and haematological malignancies together. These patient groups are significantly different and a subgroup analysis should be performed.

We appreciate the suggestion to explore whether the effect of AMR on mortality differs by type of malignancy. While we considered performing a stratified analysis, we opted instead to test for statistical interaction by including an interaction term between AMR and cancer type in a weighted Cox proportional hazards model, using the same inverse probability weights as in the main analysis. This approach maintains the structure of our existing modelling framework while allowing for a formal test of heterogeneity. The interaction term was not statistically significant ($p = 0.45$), suggesting no evidence that the association between AMR and 30-day mortality differs by cancer type. We therefore did not proceed with subgroup analyses, as this would add complexity without clear benefit.

VERSION 2 - REVIEW

Reviewer **1**

Name **Amanati, Ali**

Affiliation **Shiraz University of Medical Sciences**

Date **30-Apr-2025**

COI

The authors have notably enhanced the manuscript's clarity and readability through their recent revisions. Although most previous concerns have been addressed, an additional improvement is advised:

-Clarify whether EUCAST breakpoints were harmonised retrospectively for the entire study period or if temporal changes were accounted for. Consider sensitivity analyses using consistent breakpoint definitions.

Reviewer **2**

Name **Baltas , Ioannis**

Affiliation **University College London Hospitals NHS Foundation Trust**

Date **28-Apr-2025**

COI

I am satisfied with the changes the authors have made. My only remaining comment is that in supplementary table 7 where the antibiograms are shown, amoxicillin-clavulanate susceptibility for E.coli should be presented, given this is a first line treatment in study centers. Additionally, flucloxacillin or other ASP suspeptibility in the Swedish Staph aureus isolates should be shown, for readers to assess MRSA prevalance in that setting.

VERSION 2 - AUTHOR RESPONSE

Reviewer: 1

The authors have notably enhanced the manuscript's clarity and readability through their recent revisions. Although most previous concerns have been addressed, an additional improvement is advised:
-Clarify whether EUCAST breakpoints were harmonised retrospectively for the entire study period or if temporal changes were accounted for. Consider sensitivity analyses using consistent breakpoint definitions.

We thank the reviewer for their constructive comment. We have clarified in the Methods section that EUCAST breakpoints in effect at the time the sample was taken were used to classify AMR phenotypes. The following sentence has been added under the “Exposure” subheading:

“The exposure variable was the AMR phenotype of the causative organism in the bacteraemia, classified according to EUCAST breakpoints in effect at the time the sample was taken.”

Reviewer: 2

I am satisfied with the changes the authors have made. My only remaining comment is that in supplementary table 7 where the antibiograms are shown, amoxicillin-clavulanate susceptibility for E.coli should be presented, given this is a first line treatment in study centers. Additionally, flucloxacillin or other ASP suspeptibility in the Swedish Staph aureus isolates should be shown, for readers to assess MRSA prevalance in that setting.

We thank the reviewer for their continued engagement and helpful suggestion. We have now added amoxicillin/clavulanate susceptibility for *Escherichia coli* from both Oxford and Oslo to Supplementary Table 7. For *Staphylococcus aureus*, isolates from both sites were screened for methicillin resistance using cefoxitin. However, due to differences in laboratory workflows and reporting practices, cefoxitin results are presented for Oslo, while flucloxacillin – the reported result in the Oxford dataset – is shown for Oxford. This has been clarified in the table legend:

“Table S7. Cumulative antibiogram of the included microbes isolated from the blood of cancer patients diagnosed in 2008-2018 at Oxford University Hospitals and Oslo University Hospital. Both laboratories used cefoxitin to screen for methicillin resistance in *S. aureus*. However, due to differences in local laboratory workflows and reporting practices, cefoxitin results are shown for Oslo, whereas flucloxacillin is presented for Oxford.”

VERSION 3 - REVIEW

**Reviewer**	**1**
**Name**	**Amanati, Ali**
**Affiliation**	**Shiraz University of Medical Sciences**
**Date**	**23-May-2025**
**COI**	

I have read the revised manuscript. I have no additional comments to add. I thank the authors for their very detailed replies to my comments.

Reviewer **2**

Name **Baltas , Ioannis**

Affiliation **University College London Hospitals NHS Foundation Trust**

Date **12-May-2025**

COI

I am satified with the changes
